# The Influence of the Aircraft Operating Fluids on the Mechanical Parameters of the Airport Surface Concrete

**DOI:** 10.3390/ma13143081

**Published:** 2020-07-10

**Authors:** Wojciech Żebrowski, Paweł Wolka, Marzena Kurpinska

**Affiliations:** 1 Astra Technologia Betonu Sp. z.o.o, 83-010 Straszyn, Poland; 2 Astra Benedykt Karczewski, 83-010 Straszyn, Poland; pawel@astra-polska.com; 3 Faculty of Civil and Environmental Engineering, Gdansk University of Technology, 80-233 Gdańsk, Poland; marzena.kurpinska@pg.edu.pl

**Keywords:** airport surface concrete, hydraulic oil, lubricating oil, concrete, operating fluids, operating liquids, cement concrete

## Abstract

The authors of the article assessed the impact of operating fluids used to service aircraft on changing mechanical parameters of cement concrete intended for airport pavement. The research concerned concrete designed with the use of CEM I 42.5N LH NA low-alkali cement, broken granite aggregate, fine washed aggregate, and admixtures. The analysis included the assessment of changes in differences in endurance parameters over various research periods of up to 140 days. The obtained results allowed to carry out statistical analysis using the student’s T-test. Research has shown a significant impact of operational fluids used in aircraft on the surface concrete properties of the airport. A reduction in the compressive strength of concrete exposed to one of the tested operating liquid to a reduction of 7.2% was observed over a period of 140 days, while there was no significant impact of operating fluids on tensile strength at splitting.

## 1. Introduction

Current and forecast air transport development trends indicate an intensive development of domestic and international infrastructure of airports. The assumed evolution of air transport services will generate a significant density of aircraft traffic at airports. The consequence of intensified, safe aircraft take-off and landing operations is the need for ensuring adequate durability and quality of concrete airport pavements. According to the literature data, research on obtaining satisfactory performance characteristics of concrete used in airport pavements boiled down to providing relatively high strength parameters with high frost resistance in the presence of de-icing agents [1]. Observations carried out in the airport pavement areas indicate the occurrence, often not taken into account, of additional impacts resulting from the operation of aircraft. One of these types of impact is the presence of operating fluids from aircraft on pavements. The visible effect of such events are surface discoloration and surface damage.

**Figure 1:**
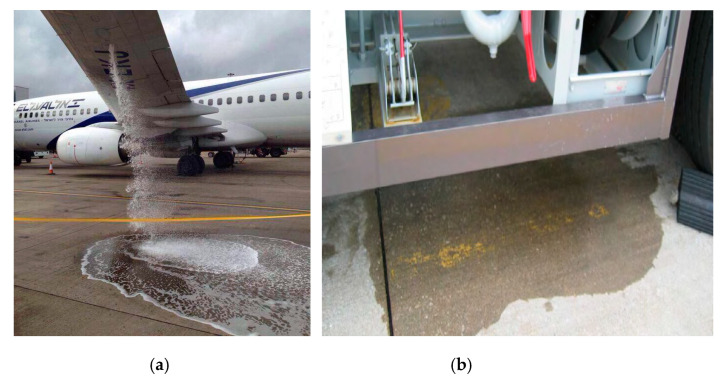
Leaking operational fluids on an airport pavement as a result of a breakdown of aircraft (**a**) and ground equipment (**b**) [2].

The presence of operating fluids on the airport pavement should be associated with their unintentional leakage from installations in the aircraft. These substances on the surface come not only from the maintenance of aircraft, aimed at maintaining the operational capacity of the aircraft, but also from the possibility of its failure and ground equipment (Figure 1). Most of the currently operated aircrafts have a number of specialized installations, which include fuel, hydraulic, oil, and pneumatic installations. Mechanical components that form complex technical systems are an integral part of the installation. These systems significantly affect the technical efficiency of the aircraft, its service life, the time it takes to prepare the aircraft for flight, and the safety of flights. They ensure interoperability of specific aircraft components. From the point of view of flight safety, aircraft systems are particularly important, as they are responsible for controlling the aircraft, in which the main energy carrier is oil in hydraulic installations.

**Figure 2:**
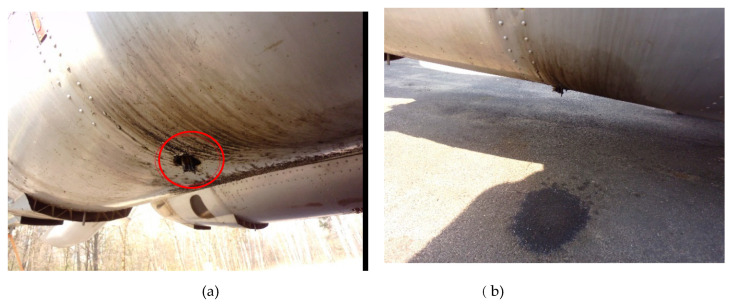
Removal of oil from the drainage installation (**a**) on the plate of airfield (**b**).

Each installation is equipped with a safety valve ended with drainage (Figure 2). Based on the experience of airport staff, it was noticed that in training flights lasting several minutes, as a result of which the aircraft repeatedly takes off and lands, from the hydraulic system drainage, during a single operation a significant amount of media is extracted onto the airport apron, e.g., in the case of Su-22 fighter it is about 2 dm^3^ of hydraulic oil. This phenomenon is related to pressure equalization in the pneumatic and hydraulic reservoir of the installation. The task of hydraulic oil in airplanes is to transfer energy in hydraulic installations as well as damping vibrations in shock absorbers and vibration dampers. 

Lubricating oil is a universal mineral oil recommended for lubrication of aviation parts requiring "light" oil, including mechanisms of pivot joints, pivot pins, and shaft joints. It is also used to secure ground equipment electronics in both civil and military aviation.

Research on the impact of operating fluids, in conjunction with the thermal effects of exhaust gases, were conducted by [3,4,5,6,7] commissioned by the US Navy, US Air Force, and the Royal Australian Air Force. However, these studies are limited in many details and are not widely published or explicitly verified.

The aim of the study was to determine the effect of hydraulic and lubricating oil on the strength characteristics of hardened concrete. Many publications indicate the need for research in this area. This issue is particularly important due to the determination of the method of maintenance and repair of surfaces of civil and military airports [8,9,10,11,12].

## 2. Theoretical Basis

Currently used operating fluids in aircraft are a complex chemical mixture. In addition to the main components of fuels and oils, "additive technology" is a separate field. This term refers to enriching additives incorporated into the operational liquids during production and storage. Contemporary mineral oils used as the main component of lubricating oils, depending on the methods of purification (refining), can be divided into conventional and unconventional.

Conventional mineral oil is obtained from crude oil in the process of solvent refining as well as acid, lye, and adsorption refining. They consist of 70–80% saturated hydrocarbons (isoparaffinic and naphthenic) and 20–30% long-chain naphthenic-aromatic and aromatic hydrocarbons. In turn, unconventional ones are obtained using catalytic processes. As a result of the processes, oils consisting of: alkylbenzenes, alkylnaphthenic, and isoparaffinic hydrocarbons are obtained [13,14]. Generally speaking, mineral oil is a mixture of hydrocarbons, which contain from 12 to 45 carbon atoms in molecules, with different structures and degrees of saturation, constituting an intermediate for further production. Due to the limited and insufficient to effectively meet the required lubricating functions in devices as well as tasks related to the protection of metal parts against corrosion and chemical instability over time, mineral oils are enriched with synthetic substances called performance additives presented in Table 1 [15].

According to [19], technical oils are more aggressive for concretes with Portland cement than with sulfate resistant cement. The authors [20] examined concretes with different water/cement ratios and treated them with mineral oil. The smallest reduction in the strength of oily concretes occurred if the w/c ≤ 0.5 index, whereas if w/c ≥ 0.6 the reduction in strength was greater than 50%. Research has shown that the type of aggregate used is significant. Concrete with limestone aggregate is much less resistant to oil than concrete with granite aggregate. According to research carried out by [21], it has been shown that the decrease in concrete strength is caused by the production of a thin insulation layer from oil. This layer insulates the cement grain against moisture and cement hydration. The hardening process stops and, as a result, the strength increases further. The age of the concrete and the duration of action of aggressive liquids are essential. Considering the system: cement stone–mineral oil, from the point of view of physical–chemical activity and the effect of reducing the strength of the hard body, we can discuss the action of wedging the structure of concrete, through a lubricating liquid moving in the capillaries. This is the so-called A. Rebinder effect [22]. In studies carried out by [23], it was shown that the decrease in strength due to the action of oil is visible after several dozen days.

**Figure 3:**
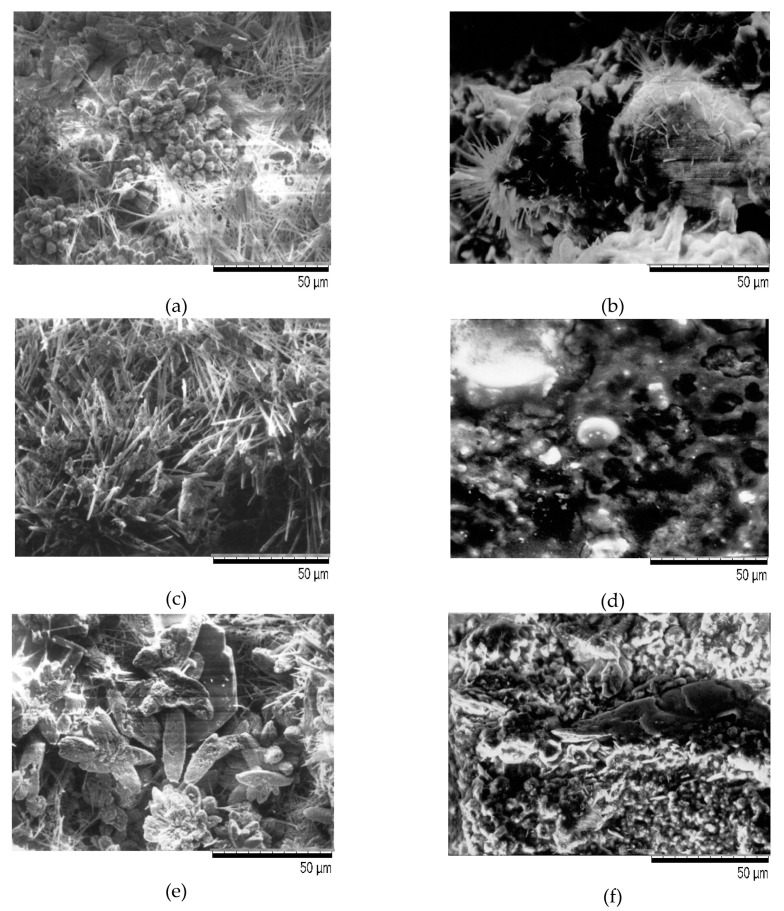
Microscopic analysis of photos of concrete samples not treated (**a**,**c**,**e**) and treated (**b**,**d**,**f**) with mineral oil.

The morphology of hydration products was assessed using a scanning electron microscope (SEM) at 2000× magnification under reduced vacuum at 60 Pa. Figure 3 shows the concrete structure of the comparative samples Figure 3a,c,e and the samples of concrete treated with mineral oil Figure 3b,d,f.

**Figure 4:**
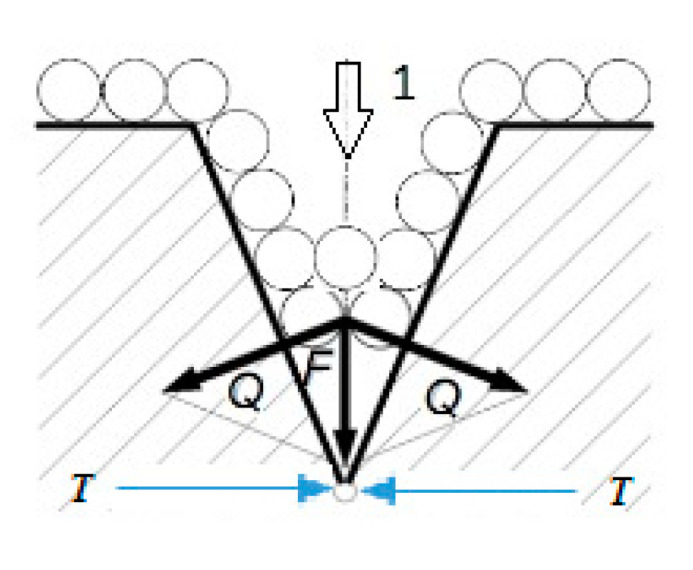
Diagram: absorption and wedging effect of oil. Where: 1—direction of liquid penetration; *Q*—wedging force; *F*—gap opening force according to the number of molecules; and *T*—molecular bond strength in a solid.

The structure of non-oiled concrete is visibly different from the structure of oily concrete. In the first case, the concrete structure is compact, the number of open pores is small, and the isomorphic structures of hydrated calcium silicates are visible–C-S-H (Calcium Silicate Hydrate) phase (Figure 3a,c) and Ca(OH)_2_–portlandite (Figure 3e). In the microscopic image, non-hydrated cement clinker grains are visible (Figure 3a), they show a transparent, weakly polarized continuous mass filled with Ca(OH)_2_ crystals and highly polarized pores evenly distributed throughout the volume (Figure 3e). In oily cross-sections, there is much less lime and calcite. In oily concrete, the structure of cement stone is changed, the pores are filled with liquid-oil (Figure 3b). In the presence of strong metal hydroxides and solutions of sodium carbonate and potassium carbonate, oil isomerization occurs. As a result of isomerization, sometimes the oils dry up and form a film (Figure 3b). Most organic substances do not react with Ca(OH)_2_. However, organic acids, as well as inorganic ones, cause acid corrosion to form calcium salts. Polyhydric alcohols, including oils, as glycerides of fatty acids may undergo hydrolysis and then decomposition into organic acids and alcohols. This happens under the influence of the alkaline environment of concrete. All products of these reactions are easily soluble, and the characteristic symptom is loosening and softening of the concrete surface [24,25], Figure 4.

## 3. Materials and Methods

### 3.1. Materials

To make concrete mixes CEM I 42.5N-MSR/NA Portland cement was used. The mentioned above cement was approved by Air Force Institute of Technology and received permission to be used in the construction of airport pavements and airport infrastructure elements. The main cement components are Portland clinker (95–56%) and a setting time regulator in the form of a mixture of anhydrite and gypsum. The Portland clinker component is a raw material with low Al_2_O_3_ content and up to 0.60% alkali Na_2_O_eq_ content, up to 7% of C_3_A tricalcium aluminate. The contents of individual cement components are presented in Table 2.

**Figure 5:**
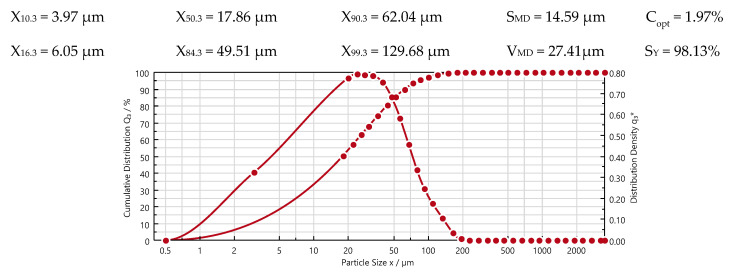
Cement grain size distribution curve CEM I 42.5 N-MSR/NA.

Particle Size and Particle Size Distribution was specified in HELOS (H3811) and RODOS/T4, R7 instrument. Grain Size distribution curve Figure 5.

**Figure 6:**
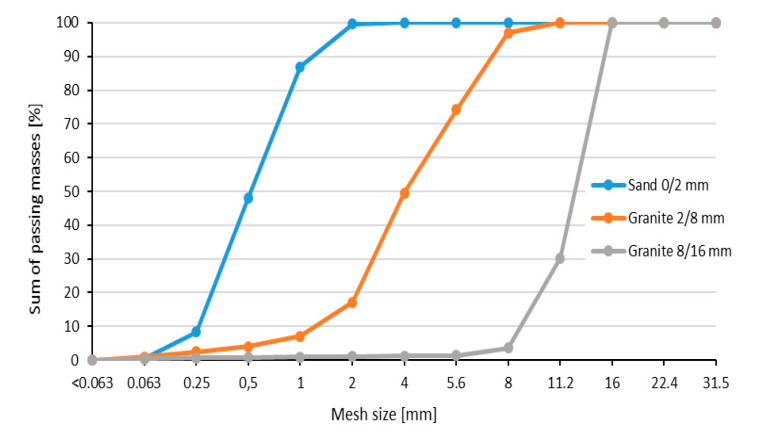
Aggregate grain distribution curves: 0/2, 2/8, and 8/16 mm.

In the concrete mixes, coarse aggregate of 2/8 mm and 8/16 mm fractions with a volume density of 2.67 kg/dm^3^ were used, derived from deep igneous rocks in the form of granite grits, in accordance with [26]. The fine aggregate for the mixes was natural sand washed with a fraction of 0/2 mm, with a density of 2.65 kg/dm^3^. The grain distribution of individual types of aggregates was made according to [27,28,29] and is presented in Figure 6.

In order to achieve good homogeneity and workability of the concrete mix, an admixture with strong plasticizing effect was used. At the same time, in order to obtain concrete resistance to aggressive atmospheric factors associated with cyclic freezing and thawing, aeration admixture was used. Drinking water from the mains was used for the tests.

### 3.2. Concrete Mix Design

**Figure 7:**
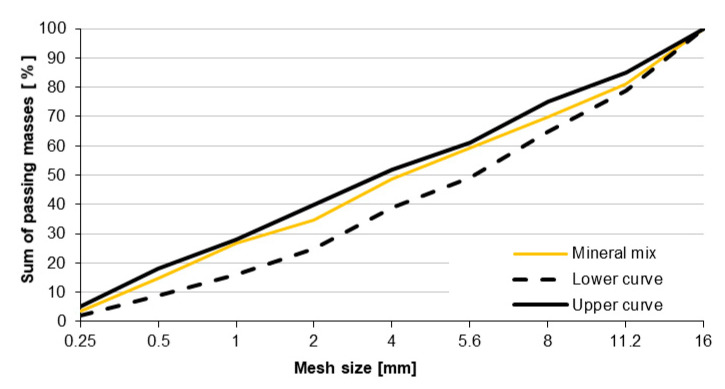
Aggregate grain size distrubution curve.

Concrete mix designs were designed in accordance with the requirements contained in [26]. Concrete strength class C30/37 was assumed. Granulation of mineral mixtures (Figure 7) was selected so that the curve of the mineral mixture remained within the field of good grain size limited by the boundary curves contained in Annex C, standards [26]. The amount of concrete mix components was determined by the analytical-experimental method, basing on the authors’ experience [28]. The included requirements of the standard [30] concerned exposure classes XC4, XF4, and XM1. Due to the impact of mineral oils on the hardened concrete, the exposure class XA2 was also taken into account. The ratio w/c = 0.4 resulting from the requirements of the standard [26] was adopted. Thanks to the use of a plasticizing admixture, the consistency of S1 (10–40mm), measured by the cone fall, was received. It enabled the surface to be made in sliding formwork. The air content of the concrete mix ranged from 4.5% to 5.5%. The volume fraction of individual components of the concrete mix is shown in Table 3, while the grain size distribution curve of the aggregate composition is shown in Figure 7.

### 3.3. Aggressive Medium

**Figure 8:**
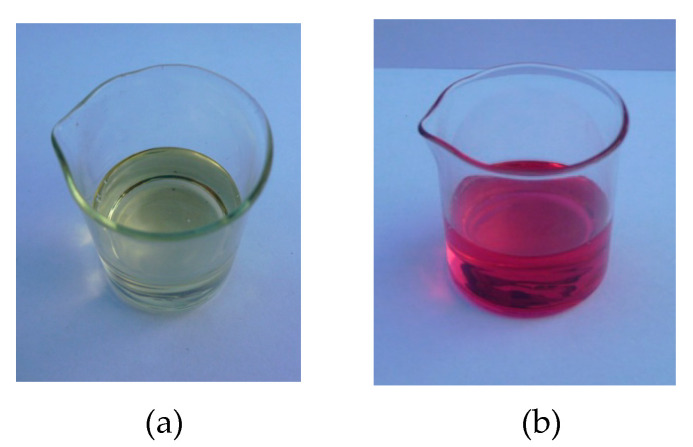
Images of (**a**) lubricating oil and (**b**). hydraulic oil.

Lubricating oil (Figure 8a) in accordance with [31] and Hydraulic mineral oil (Figure 8b) in accordance with [32] were used as the aggressive medium in the tests.

Table 4 presents the basic parameters of operating fluids used in the study.

### 3.4. Research Methodology

#### 3.4.1. Test Samples Conditioning

The process of research samples conditioning was divided into 3 parts. Initially, the formed samples were stored in molds for 24 h, protected with foil against moisture loss. Subsequently to day 28, the samples were conditioned according to the standard [30] in water at 20 °C ± 2. After the standard conditioning period, the samples were left in air-dry conditions for 14 days. Ultimately, the samples were divided into control concrete samples (witnesses) and samples for testing the impact of hydraulic liquids. Research periods were adopted as a multiple of the 28-day study period.

#### 3.4.2. Compressive Strength

The compressive strength test was carried out on six cubic samples 150 mm × 150 mm × 150 mm prepared in accordance with [33], control concrete and concrete exposed to a given oil based on the standard procedure [34]. The strength press Controls MMC8 in accordance with [35] was used for strength tests. The prepared sample for testing was placed centrically between the pressure plates in such a way that the load was oriented perpendicularly to the direction of sample formation. The load speed was set at 0.5 MPa/s and the test continued until the sample was destroyed [36]. Compressive strength was calculated from formula (1).

(1) $$f_{c}=\frac{F}{A_{c}}$$

where: $f_{c}$—compressive strength, in MPa, *F*—maximum load at failure, in kN, and $A_{c}$—transverse surface area of the sample calculated on the basis of measurements in mm^2^.

#### 3.4.3. Tensile Strength at Splitting

Concrete tensile strength testing using the indirect method was carried out in accordance with [37] on six 150 mm × 150 mm × 150 mm samples. The test was performed on samples of control concrete and concrete exposed to operating fluids. The prepared sample was placed centrally in a steel frame with curved loading elements, between standard spacers. The stress increase was set at 0.05 MPa/s. The study was conducted until the sample was destroyed by stretching. Tensile strength at splitting was calculated according to formula (2):

(2) $$f_{ct}=\frac{2\cdot F}{\pi \cdot L\cdot d}$$

where: *f*_ct_—tensile strength at splitting, in MPa; *F*—maximum load at destruction, in N; *L*—length of sample contact line, in mm; and *d*—cross-sectional dimension, in mm.

## 4. Results and Discussion

### 4.1. Compressive Strength

**Figure 9:**
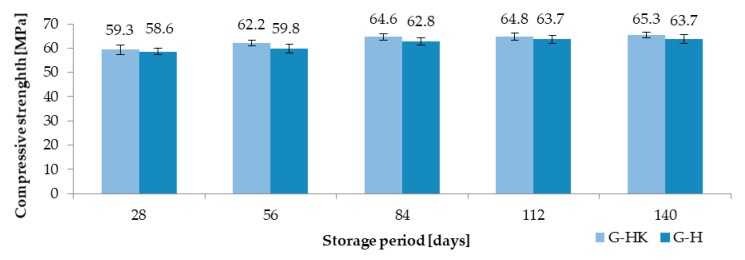
Compressive strength results of concrete exposed to hydraulic oil (G-H) and control concrete (G-HK).

The average results of the compressive strength of concrete with granite aggregate exposed to hydraulic oil (GH) and control concrete stored in air-dry conditions (G-HK) are summarized in Figure 9. The control concrete was characterized by a higher compressive strength compared to the concrete exposed to the medium operation in each research period. As a result of the impact of hydraulic oil, a reduction in strength of 1.2% (28 days), 3.8% (56 days), 2.8% (84 days), 1.7% (112 days), 2.4% (140 days), and 3.7% (168 days) was observed.

**Figure 10:**
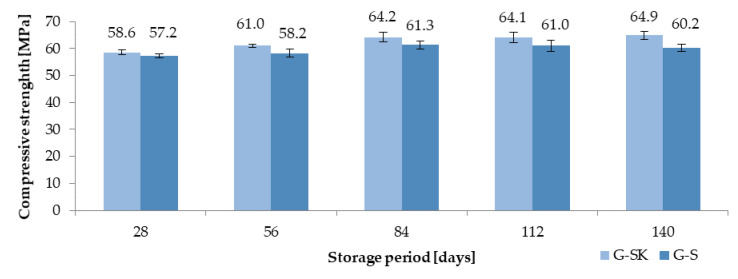
Compressive strength results of concrete exposed to lubricating oil (G-S) and control concrete (G-SK).

Similar relationships were observed in the case of the lubricating oil interaction. They are shown in Figure 10. Concrete exposed to lubricating oil (GS) was characterized by reduced strength compared to the control concrete (G-SK), respectively: 2.4% (28 days), 4.6%, (56 days), 4.5% (84 days), 4.8% (112 days), 7.2% (140 days), and 7.2% (168 days).

### 4.2. Tensile Strength When Splitting

**Figure 11:**
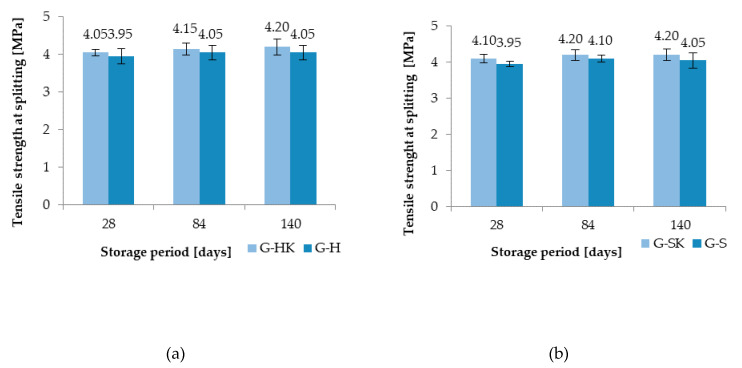
Results of tensile strength when splitting concrete exposed to (**a**) hydraulic oil (G-H) and (**b**) lubricating oil (G-S) compared to control concrete (G-HK, G-SK).

The average results of tensile strength at splitting concrete with granite aggregate exposed to hydraulic oil (GH) and control oil (G-HK) stored in air-dry conditions during various testing periods are shown in Figure 11a. The reduction in strength compared to control concrete respectively: 2.5% after 28 days, 2.4% after 84 days, and 3.6% after 140 days of hydraulic oil exposure was observed.

Figure 11b shows the average results of tensile strength when splitting concrete with granite aggregate exposed to lubricating oil (G-S) and control oil (G-SK) stored in air-dry conditions during various test periods. As a result of the action of lubricating oil, a reduction in strength compared to the control concrete was achieved respectively: 3.7% after 28 days, 2.4% after 84 days, and 3.6% after 140 days.

**Figure 12:**
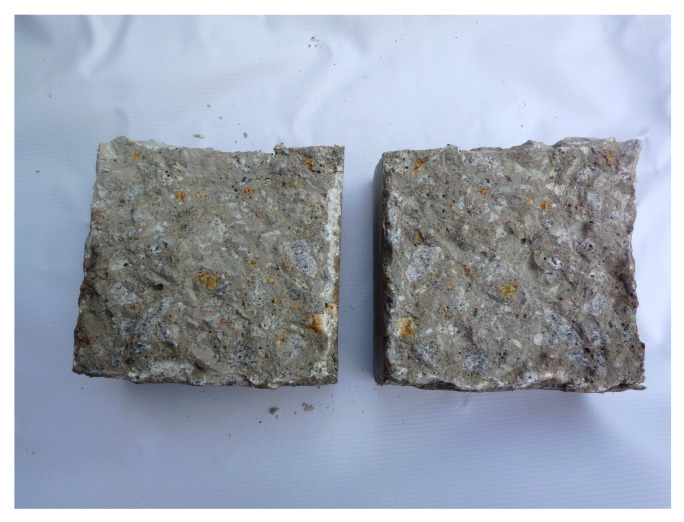
Fracture sample treated the lubricating oil.

View of samples after the tensile strength when splitting test is shown in Figure 12.

### 4.3. Statistical Analysis

In order to verify the obtained test results, a statistical analysis was performed by means of Student’s t-test and analysis of post-hoc variance with Fisher’s LSD (Least Significant Differences) test using the R program [38]. The level of significance 0.05 was adopted in the analyses. All *p* < 0.05 values were interpreted as indicating significant relationships. The t-Student test was used to compare the results obtained for concrete in two groups-concrete exposed to a given medium and control concrete. The t-Student test for independent groups was chosen because separate results were obtained for the analyzed concrete groups. The null hypothesis was adopted: the average results of concrete exposed to the operating medium are equal to the average results of the control concrete and the alternative hypothesis: the average results of the concrete exposed to the operating medium differ from the average results obtained for the control concrete. The basis for statistical analysis were the assumptions according to [39]:

- the distribution of results in each of the analyzed concrete groups is comparable to the normal distribution;
- compared groups of concretes are equal;
- variances in the compared groups are similar; and
- the dependent variable is expressed on a quantitative scale.

The statistics used were according to formula (3):

(3) $$t=\frac{{\bar{X}}_{1}-{\bar{X}}_{2}}{2\sqrt{s_{1}{}^{2}-s_{2}{}^{2}}}\cdot \sqrt{n-1}$$

where: ${\bar{X}}_{1,2}$—average in individual samples, *s_1,2_^2^*—variance from individual trials, *n*—number of attempts.

In Table 5, detailed results for individual concrete groups exposed to operating fluids are listed together with significance level (*p*) values.

In the statistical T-student test a significance level of p = 0.05 was assumed. The test showed that the differences between the strength of concrete exposed to operating fluids and control concrete are statistically significant in selected research periods. In the case of the compressive strength test, statistically significant differences occurred after 140 days of hydraulic oil exposure. However, the examined feature in the case of lubricating oil was statistically significant in all research periods.

The impact of operating fluids on the examined tensile strength feature during splitting in the assumed research periods did not show statistically significant differences.

### 4.4. Comparasion of Results and Interpretations with Those of Other Workers

Considering operating fluids used in aircrafts reduce the tested strength parameters of hardened concrete, the negative impact is particularly evident during the period of prolonged conditioning. The observed decrease in strength after 140 days of the lubricating oil effect on cement concrete, amounting to 7.2%, shows the significance of the problem posed in the article. Similar studies on the impact of substances produced from crude oil processing, related to structural elements made of cement concrete were conducted by the authors [40,41,42,43,44,45,46,47,48,49,50,51,52,53,54,55]. They present physical, chemical, and physical–chemical mechanisms as the reasons for the decrease in concrete strength [40,43]. The authors used liquids with an acid number <0.25 mg KOH/g for testing, therefore, basing on the authors’ experience [40,41,42,43], the possibility of chemical degradation of the concrete structure was excluded. The likely decrease in strength parameters should be associated with the presence of surfactants in the composition of oils. The action of surfactants in the structure of concrete should be associated with the possibility of physical and chemical phenomena leading to concrete degradation. These include the following: reduction of surface energy in the concrete structure by adsorption of the interacting substance, partial stopping of the hydration process as a result of concrete hydrophobization, and the presence of polar particles weakening the binding of the hardened composite. However, the presence of oil in the concrete pore structure has a positive effect on increasing the modulus of elasticity. Runkiewicz et al. [55] showed that the coefficient of elasticity of concrete is greater until the liquid is squeezed out of the pores. The resistance of the liquid before pushing it out of the pores reduces the deformation. A broader explanation of the processes taking place in the internal structure of concrete requires further research.

## 5. Conclusions

The presented research was conducted to evaluate the influence aircraft operating fluids on the parameters of hardened concrete with granite coarse aggregate. Laboratory results have made it possible to draw the following conclusions:

- In the case of their leakage on the airport apron, the operational liquids used in tests for civil and military aircraft have a significant impact on the strength properties of concrete and its durability.
- Depending on the type and properties of oils, their aggressiveness towards concrete varies. It depends on the chemical activity of fatty acids and glycerin contained in oils, in relation to the alkaline environment of the calcium hydroxide solution in the pores of hardening cement stone. The degree of aggressiveness determines the reduction of concrete strength at the initial stage of hardening.
- The short-term effect of oils in many cases can result in hydrophobization of concrete and, thus, increase its corrosion resistance, water resistance, and frost resistance. However, this phenomenon will depend on the type and composition of the oil. Oil-resistant concrete with a tight structure, with closed pores and a minimum content of soluble calcium hydroxide will be resistant to oil. For such concretes it is recommended to use special cements, e.g., resistant to sulfates and calcium hydroxide binding additives.
- Long-lasting hydraulic and lubricating oil have a destructive effect on concrete. A reduction in the compressive strength of concrete exposed to one of the tested media to 7.2% was observed over a period of 140 days.
- Statistical analysis using the T-student test showed the significance of differences between the parameters of the compressive strength of the concrete exposed to the effect of lubricating oil in each test period. On the other hand, no significant impact of operating fluids on tensile strength when cracking surface concrete was observed in all test periods (*p* > 0.05).
- The presented test results indicate a destructive impact of operating liquids on concrete airport pavements. Given the above, and taking into account the need to ensure the safety of air operations, the need to develop:
  ○ preparations characterized by increased effectiveness in securing the surface layer of cement concrete exposed to the effects of operating fluids;
  ○ selection of the type and number of components, concrete should be characterized by very good adhesion in the aggregate-slurry zone. It is possible to do by modifying the composition of the concrete mix with mineral additives, e.g., based on aluminosilicates [52];
  ○ in a situation of permanently changing properties of liquids used in the operation of aircrafts and technology, conditions for the preparation and incorporation of surface concrete must be carried out at the highest level, taking into account stringent requirements.

## Figures and Tables

**Table 1:** Nature of the action of additives in mineral oils [16,17,18].

**Chemical**	**Interphase**	**Volume**
	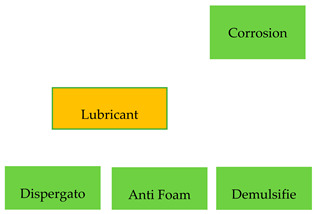	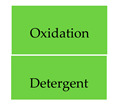
**Physical**		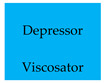
**Nature of action**	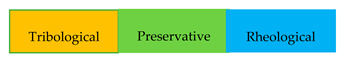	

**Table 2:** Chemical composition of CEM I 42.5 N-MSR/NA cement.

Material	Content (%)									
	SiO_2_	Al_2_O_3_	Fe_2_O_3_	CaO	MgO	SO_3_	Na_2_O_eq_	Cl^-^	Loss on Ignition	Other
**Cement**	21.0	3.64	3.13	66.03	0.79	2.51	0.39	0.05	2.10	0.36

**Table 3:** Concrete mix design with aggregate composition.

Ingredient	Density (kg/dm^3^)	Percentage (%)
**Cement**	3.10	13.73
**Sand 0/2 mm**	2.65	21.81
**Granite 2/8 mm**	2.67	34.73
**Granite 8/16 mm**	2.67	24.24
**Water**	1.00	5.49
**Plast. [%] c.c.**	1.14	1.10
**Air LP [%] c.c.**	1.00	0.50

**Table 4:** Basic physical characteristics of hydraulic and lubricating oil.

Oil Type	Kinematic Viscosity in 20 °C (mm/s^2^)	Density in 15 °C (kg/m^3^)	Acid Value (mg KOH/g)
Hydraulic Oil	13	870	<0.20
Lubricating Oil	9	870	<0.04

**Table 5:** The significance of differences between the mechanical parameters of concrete exposed to operating fluids (G-H, G-S) and control concrete (G-HK, G-SK).

Compressive Strength					
Days		G-H	G-HK	G-S	G-SK
28	Average ± SD	58.57 ± 1.16	59.28 ± 2.12	57.22 ± 0.83	58.6 ± 0.88
	t-Student	p = 0,484		p = 0,019	
56	Average ± SD	59.8 ± 1.85	62.15 ± 1.11	58.18 ± 1.54	61.03 ± 0.67
	t-Student	p = 0.092		p = 0.002	
84	Average ± SD	62.78 ± 1.63	64.63 ± 1.33	61.32 ± 1.45	64.2 ± 1.78
	t-Student	p = 0.057		p = 0.012	
112	Average ± SD	63.68 ± 1.72	64.82 ± 1.54	60.95 ± 2.15	64.1 ± 2.05
	t-Student	p = 0.256		p = 0.027	
140	Average ± SD	63.63 ± 1.91	65.35 ± 1.2	60.23 ± 1.42	64.9 ± 1.49
	t-Student	p = 0.024		<0.001	
**Tensile Strength at Splitting**					
28	Average ± SD	3.94 ± 0.09	4.03 ± 0.08	3.95 ± 0.08	4.11 ± 0.12
	t-Student	p = 0.097		p = 0.124	
84	Average ± SD	4.03 ± 0.2	4.14 ± 0.16	4.1 ± 0.1	4.18 ± 0.13
	t-Student	p = 0.286		p = 0.24	
140	Average ± SD	4.07 ± 0.1	4.17 ± 0.22	4.03 ± 0.21	4.2 ± 0.17
	t-Student	p = 0.326		p = 0.15	

## References

[1] Neville A.M. (2011). Properties of Concrete.

[2] Smith H. Safe Operation of Vehicles and Personnel in Movement and Safety Areas. Present at Fueling safety, Aerodrome Inspectors Workshop, Federal Aviation Administration.

[3] Duane B.W. (2016). The behaviour of airfield rigid pavements under the influence jet fuel, lubricating and hydraulic fluids and cyclic heat loading by F/A-18 APU Exhaust. Bachelor’s Thesis.

[4] Malvar L.I., Hironaka M.C. (1991). Heat resistant concrete for airfield pavements: Preliminary numerical study. Comput. Struct..

[5] McVay M., Rish J., Sakezless C., Mohsen S., Beatty C. (1995). Cements resistant to synthetic oil, hydraulic fluid and elevated temperature environments. ACI Mater. J..

[6] McVay M.C., Smothson L.D., Manzione C.H. (1993). Chemical damage to airfield concrete aprons form heat and oils. ACI Mater. J..

[7] Shill S.K., Al-Deen S., Ashraf M. (2018). Concrete durability iss. due to temperature effects and spillage at military airbase – A comprehensive review. Constr. Build. Mater..

[8] Linek M., Nita P., Żebrowski W., Wolka P. (2019). Influence of operating media on the parameters of cement concrete intended for airfield pavements. J. KONBIN.

[9] Linek M. Modified Pavement Quality Concrete as Material Alternative to Concrete Applied Regularly on Airfield Pavements. Present at WMCAUS 2019.

[10] Linek M., Żebrowski W., Wolka P. (2016). Change of physical parameters and compressive strength of airport concrete under the influence of hydraulic mineral oil. Build. Mater..

[11] Nita P. (1986). Some properties of surface concretes treated chemicals occuring in pavement exploitation process. Road Constr..

[12] Wisconsin Bureau of Aeronautics, State of Wisconsin Department of Transportation Bureau of Aeronautics (1998). Standard Spacifications for Airport Construction.

[13] Nowak P., Kucharska K., Kamiński M. (2019). Ecological and health effects of lubricant oils emitted into the environment. Int. J. Environ. Res. Public Health.

[14] Mortier R.M., Orszulik S.T. (1997). Chemistry and Technology of Tubricants.

[15] Zwierzycki W. (1999). Industrial Oils and Greases, Instytut Technologii.

[16] Minami I. (2017). Molecular science of lubricant additives. Appl. Sci..

[17] Papay A.G. (1998). Antiwear and Extreme-Pressure Additives in Lubricants. Lubr. Sci..

[18] Rizvi S.Q.A. (1999). Additives for Automotive Fuels and Lubricants. Lubr. Eng..

[19] Garboczi E.J. (1990). Permeability, diffusivity and microstructural parameters: A critical review. Cem. Concr. Res..

[20] Watson A.J., Oyeka C.C. (1981). Oil Permeability of Hardened cement Paste and Concrete. Mag. Concr. Res..

[21] Васильев Н.М. (1981). The effect of petroleum products on the strength of concrete. Concr. Reinf. Concr..

[22] Osuji S., Nwankwo E. (2015). Effect of Crude Oil Contamination on the Compressive Strength of Concrete. Niger. J. Technol..

[23] Wolicka D. (2010). Microorganisms found in crude oil and in reservoir waters. Oil Gas.

[24] Salman A.A.A., Alghazali J.J.H., Alwash N.O.S. (2018). The effect of fibers on the properties of self-compacting concrete subjected to petroleum products. Int. J. Civ. Eng. Technol..

[25] Svinstov A.P., Nikolenko Y.V., Kharun M., Kazakov A.S. (2014). Effect of viscosity of petroleum products on deformation properties of concrete. Mag. Civ. Eng..

[26] NO-17-A204: 2015 (2015). Airport Pavements. Cement Concrete Surfaces. Requirements and Test Methods.

[27] Linek M., Nita P., Wolka P., Żebrowski W. Usefulness of porphyry and amphibolites as a component of concrete for airfield pavements. Present at MATBUD’2018—8th Scientific-Technical Conference on Material Problems in Civil Engineering.

[28] Linek M., Nita P., Żebrowski W., Wolka P. Assessment of Granite, Quartz and Syenite Aggregate Suitability Intended for The Application in Case of Transport Pavement Concrete. Present at WMCAUS 2018.

[29] BS EN 933-1:1997 (1997). Tests for Geometrical Properties of Aggregates–Part 1. Determination of Particle Size Cistribution–Sieving Method.

[30] BS EN 206:2013+A1:2016 (2016). Concrete. Specification, Performance, Production and Conformity.

[31] MIL-PRF-7870D (2010). Performance Specification. Lubricating Oil: General Purpose, Low Temperature (NATO O-142).

[32] MIL–PRF–5606H (2002). Performance Specification. Hydraulic Fluid, Petroleum Base, Aircraft, Missile, and Ordance.

[33] BS EN 12390-2 Pdf standard (2000). Testing Hardened Concrete–Part 2–Making and Curing Specimens for Strength Tests.

[34] CEN 12390-1 (2012). Testing Hardened Concrete—Part 1: Shape, Dimensions and Other Requirements for Specimens and Moulds.

[35] BS EN 12390-4:2019 (2019). Testing Hardened Concrete. Compressive Strength. Specification for Testing Machines.

[36] BS EN 12390-3 (2009). Testing Hardened Concrete—Part 3: Compressive Strength of Test Specimens.

[37] BS EN 12390-6:2009 (2009). Testing Hardened Concrete. Tensile Splitting Strength of Test Specimens.

[38] R Core Team (2017). A Language and Environment for Statistical Computing.

[39] Benjamin J., Cornell C. (1970). Probability Statistics and Decision for Civil Engineers.

[40] Błaszczyński T. (2011). Assessment of RC structures influenced by crude oil products. Archives Civ. Mech. Eng..

[41] Osial M., Wiliński D. (2016). Organic substances as corrosion inhibitors for steel in concrete–an overview. J. Build. Chem..

[42] Smith J.L., Virmani Y.P. (2000). Materials and Methods for Corrosion Control of Reinforced and Prestressed Concrete Structures in New Construction.

[43] Błaszczyński T.Z. (2011). The influence of crude oil products on RC structure destruction. J. Civ. Eng. Manag..

[44] Diab H. (2012). Compressive strength performance of low-and high strength concrete soaked in mineral oil. Constr. Build. Mater..

[45] Boos P., Giergiczny Z. (2010). Testing the frost resistance of concrete with different cement types—Experience from laboratory and practice. Archit. Civ. Eng. Environ..

[46] Gruszczyński M., Lenart M. (2019). Liquid penetration depth and strength of concretes modified with polymer admixtures under the action of crude-oil products. Materials.

[47] Hamad B.S., Rteil A.A. (2003). Effect of used oil on structural behavior of reinforced concrete elements. Construction and Building. Materials.

[48] Hamad B.S., Rteil A.A., El-Fadel M. (2003). Effect of used engine oil on properties of fresh and hardened concrete. Constr. Build. Mater..

[49] Kamaitis Z. (2009). Modelling of corrosion protection as standby system for coated reinforced concrete structures. J. Civ. Eng. Manag..

[50] Kurpińska M. (2011). Properties of concrete impregnated using epoxy composition. Roads Bridges.

[51] Svintsov A.P. (2019). Effect of petroleum products on physical and mechanical properties of concrete and the reliability of load-bearing structures. Arab. J. Sci. Eng..

[52] Mariak A., Kurpińska M. The effect of macro polymer fibres length and content on the fibre reinforced concrete. Present at 2nd Baltic Conference for Students and Young Researchers.

[53] Golewski G.L. (2019). Estimation of the optimum content of fly ash in concrete composite based on the analysis of fracture toughness tests using various measuring systems. Constr. Build. Mater..

[54] Golewski G.L. (2019). The influence of microcrack width on the mechanical parameters in concrete with the addition of fly ash: Consideration of technological and ecological benefits. Constr. Build. Mater..

[55] Runkiewicz L., Konieczny K., Brzęk R. (2002). Changes in strength and deformability of oily concrete in structures. Constr. Rev..

